# Cryogenic Insulation—Towards Environmentally Friendly Polyurethane Foams

**DOI:** 10.3390/polym16172406

**Published:** 2024-08-24

**Authors:** Laima Vevere, Vladimir Yakushin, Beatrise Sture-Skela, Janis Andersons, Ugis Cabulis

**Affiliations:** Latvian State Institute of Wood Chemistry, 27 Dzerbenes Str., LV-1006 Riga, Latvia; laima.vevere@kki.lv (L.V.); vladimir.yakushin@kki.lv (V.Y.); beatrise.sture@kki.lv (B.S.-S.); janis.andersons@pmi.lv (J.A.)

**Keywords:** energy saving, liquid gasses storage, space technologies, fourth-generation blowing agents, bio-based polyols

## Abstract

Cryogenics is the science and technology of very low temperatures, typically below 120 K. The most common applications are liquified natural gas carriers, ground-based tanks, and propellant tanks for space launchers. A crucial aspect of cryogenic technology is effective insulation to minimise boil-off from storage tanks and prevent frost build-up. Rigid closed-cell foams are prominent in various applications, including cryogenic insulation, due to their balance between thermal and mechanical properties. Polyurethane (PU) foam is widely used for internal insulation in cryogenic tanks, providing durability under thermal shocks and operational loads. External insulation, used in liquified natural gas carriers and ground-based tanks, generally demands less compressive strength and can utilise lower-density foams. The evolution of cryogenic insulation materials has seen the incorporation of environmentally friendly blowing agents and bio-based polyols to enhance sustainability. Fourth-generation physical blowing agents, such as HFO-1233zd(E) and HFO-1336mzz(Z), offer low global warming potential and improved thermal conductivity. Additionally, bio-based polyols from renewable resources like different natural oils and recycled polyethylene terephthalate (PET) are being integrated into rigid PU foams, showing promising properties for cryogenic applications. Research continues to optimise these materials for better mechanical performance and environmental impact.

## 1. Introduction

Cryogenic insulation is an indispensable and efficient measure to counteract undesirable heat gain, conserve energy, and prevent the boiling of gases at extremely low temperatures. Its application extends from cold storage units and liquefied natural gas carriers to the insulation of cryogenic propellant tanks for space launchers. Choosing an appropriate insulation material for cryogenic purposes can be challenging, as materials that excel as insulators at room temperature may not perform similarly at cryogenic temperatures. The selected cryogenic insulation must have low thermal conductivity, high mechanical strength, good adhesion to the base material, and endure mechanical stresses without compromising its insulating capabilities. For liquified gas carriers and space launchers, cryogenic insulation also has to be lightweight, as any additional weight increases the fuel consumption of the vehicle.

The European Union wants to reach climate neutrality by 2050. To reach this goal, all economic sectors need to decarbonise. Better insulation, including cryogenic insulation, saves energy. Promising technologies like hydrogen-based aviation fuels are anticipated to gradually contribute to the decarbonisation of air travel, starting with short-haul flights, as stated in EU directive 2018/2001 [1]. Also, the International Civil Aviation Organisation, in the 41st Assembly, adopted a long-term global aspirational goal for international aviation of net-zero carbon emissions by 2050 [2]. They also include cryogenic hydrogen as a fuel option for aircraft. This means cryogenic insulation will become even more important in the coming decades.

Several materials are used for cryogenic insulation: aerogels, rigid polyurethane (PU) foams, and multilayer insulation (MLI). Vacuum-based MLI possesses the lowest thermal conductivity among them, within 0.01 to 1 mW/(m·K) range [3,4], but its application is hampered by the need to maintain vacuum, which entails heavier tank walls and also the risk of catastrophic failure in thermal insulation upon loss of vacuum [5,6]. This risk can be mitigated by using composite insulation consisting of PU foams and MLI [7,8]. The thermal resistivity of vacuum-based aerogels is also higher than that of PU foams, but they exhibit roughly comparable thermal conductivity at ambient pressure [9,10]. Moreover, rigid PU foams have one major benefit over other materials, which is their ability to be sprayed onto complicated-shaped surfaces. While seams and joints, which result from the specific installation techniques of other insulation materials, may result in thermal leaks, such structural weak spots are largely absent in spray-on PU foam insulation (SOFI). Moreover, the adhesive bond forming between SOFI and the cryogenic tank eliminates the need to apply an adhesive. Comparison between main types of cryogenic insulation are shown in Table 1.

The demand for efficient cryogenic insulation is increasingly being driven by the necessity of more sustainable development of air, sea, and land transportation. Growing emphasis on renewable energy sources entails a drastic reduction in the consumption of fossil fuels. A possible low-emission replacement for fossil fuels is hydrogen [11], the most efficient utilisation of which for heavy-duty vehicles is in the form of liquid hydrogen (LH_2_), necessitating cryogenic insulation of its storage tanks [12]. LH_2_ has long been considered an aircraft fuel, and interest in its application in civil aviation has renewed lately [13]. LH_2_ tanks are being developed for aviation [14,15,16,17], marine [18], and railway [19] transport. Along with effective insulation, a sufficient extraction rate of H_2_ gas during the operation of the vehicle engine may need to be ensured. To ascertain an adjustable boil-off rate of LH_2_ with reduced active heating, inserting an intermediate layer into the PU foam insulation of the aircraft cryotank has been proposed [17]. This layer can be filled with liquid nitrogen while on the ground, facilitating active cooling, and flooded with ambient air during flight for passive heat input [17].

Thus, efficient cryogenic insulation can enable greener transport and cryo-storage industries. Another aspect of sustainability concerns reducing the environmental footprint of the cryogenic insulation materials themselves. In the case of PU foams, this can be attained by increasing the fraction of sustainably sourced and/or environmentally friendly components used in their production. This review considers the application of PU SOFI in cryogenics and the possibilities of their sustainable production and environmentally friendly exploitation. This paper is organised as follows: Section 2 presents a review of the cryogenic application of rigid polyurethane foams produced from commercially available polyols, Section 3 considers the application of environmentally friendly blowing agents in production in PU SOFI, and Section 4 discusses the utilisation of bio-based polyols in cryogenic rigid PU SOFI. In this review, it has been confirmed that novel polyols and environmentally friendly physical blowing agents make it possible to obtain competitive rigid PU foams for next-generation green, effective energy storage and transport.

## 2. Cryogenics and Cryogenic Rigid Polyurethane Foams from Commercially Available Polyols

Cryogenics means the science and technology of very low temperatures. The 13th IIR International Congress of Refrigeration (held in Washington DC in 1971) endorsed a universal definition of “cryogenics” and “cryogenic” by accepting a threshold of 120 K (or −153 °C) to distinguish these terms from conventional refrigeration [20,21,22]. This is a logical dividing line since the normal boiling points of the so-called permanent gases (such as methane, nitrogen, oxygen, normal air, argon, neon, hydrogen, and helium) lie below 120 K, while the freon refrigerants, hydrocarbons, and other common refrigerants have boiling points above 120 K.

An obligatory element of cryogenic technology is cryogenic insulation. It is installed both on vessels for the storage and transportation of liquefied gases and on pipelines for pumping out and handling devices. It should reduce the amount of boil-off from the storage tanks and prevent frost build-up on the outer surface of the tanks and pipelines [23,24,25]. Cryogenic insulations can be divided into two basic insulation types: vacuum and massive (solid materials with low thermal conductivity, like MLI and rigid PU foams). Vacuum insulation systems are generally used on piping and vessels containing liquid helium or hydrogen. They consist of highly polished metal supporting walls with a vacuum space between them. Frequently, multiple metal reflective foils or opacified powders are placed between the walls to reduce the radiative heat transfer further [26].

The best possible insulation would be granted with a high vacuum with MLI [27,28,29]. The thermal conductivity of aerogel bead, perlite, and glass microspheres in a high vacuum is higher. Basic heat transfer mechanisms and existing experimental data of evacuated multilayer insulation and different kinds of evacuated powder insulation, including metallised hollow microspheres, were studied by Cunnington, Fesmire, and others [3,30,31,32,33]. The MLI is the most efficient insulation, but it has some serious drawbacks; in the case of damage, MLI loses all insulation properties, and it is hard to fix.

The design of the cryogenic liquid gas tanks requires a carefully balanced compromise between mechanical and thermal requirements, gravimetric storage density, and cost-effectiveness. In many cases, due to the consideration of all these factors, despite the much lower thermal conductivity of the MLI, the second type of insulation, a rigid closed-cell foam, is used to prevent cryo-pumping [14]. Depending on the tank’s design, cryogenic insulation can be internal or external [15]. In any case, the insulation must withstand not only thermal stresses but also other operational loads.

Internal cryogenic insulation was used in the design of the liquid hydrogen tank in the first three stages of the Saturn V Launch Vehicle. It was capable of withstanding the thermal shock associated with the loading of LH_2_ and providing adequate insulative properties to limit the flow of heat into the LH_2_. It also withstands tank-filling liquid hydrogen pressure. For this, polyurethane foam, reinforced in all three planes with fibreglass threads, was used [34,35,36].

The feasibility of the application of reinforced rigid PU foam in the design of common bulkheads for cryogenic tanks is analysed in [37]. The common bulkhead of tanks for two liquid gases effectively reduces the structural weight and improves the structural performance. It is a sandwich structure filled with foam between two alloy panels of aluminium–lithium for thermal insulation. Reinforcing the foam was necessary to achieve the required strength and compressive stability. For the PU foams with a density of 43 kg/m^3^, the compression strength was 0.33 MPa, the yielding strength was 0.32 MPa, and the elasticity modulus was 32 MPa.

The most widely reinforced rigid PU foam is used as the internal cryogenic insulation of liquefied gas carriers’ membrane tanks of the GTT Mark III design concept (GTT Training Ltd.). The insulation system is comprised of three layers glued together: a secondary polyurethane foam panel reinforced with a fibreglass mat glued to a plywood board, a composite material layer that acts as a secondary barrier, and the primary polyurethane foam layer reinforced with a fibreglass mat attached to a plywood board. The insulation panels are glued to the inner hull of the carrier with mastic ropes that also function to support the insulation and the membranes. Additionally, the mastic ropes compensate for any inner hull unevenness [38,39,40,41].

A feature of the internal insulation of liquified natural gas (LNG) carrier tanks is that, in addition to thermal and static compression stress stability, it requires structural stability under impact loads, such as sloshing [42]. The properties of various reinforced polyurethane foams intended for the internal insulation of LNG tanks and their behaviour at cryogenic temperatures are considered in many works [43,44,45,46,47,48,49,50]. Some of them also studied the behaviour of these foams under impact loads [51,52]. In addition to traditional glass fibres, aramid and carbon nanotubes have also been tried as reinforcement [53,54,55]. Analysis of stresses and cryogenic reliability of composite insulation panels for LNG ships is considered in the following works [56,57,58].

Quite different requirements are imposed on external cryogenic insulation. Moreover, depending on the operating conditions, they can be completely different. However, in most cases, the requirements for compressive strength are not as high as those for internal insulation. Therefore, a set of requirements for external insulation can be met by foam plastics of much lower density (35–55 kg/m^3^) than the usual density of internal insulation (100–140 kg/m^3^).

External polymer foam insulation is used in LNG carriers with independent tanks of type B systems. They are spherical Moss tanks and self-supporting prismatic tanks by IHI Corporation. A standard Moss insulation system consists of multiple layers of extruded or expanded polystyrene with an aluminium foil vapour barrier on the outside. Typically, glass fibre mesh cloth reinforcement is glued between layers in the insulation. Another insulation system used for Moss LNG carriers uses rigid PU foam panels, polystyrene panels, or a composite panel system fabricated from a layer of polyurethane foam, a steel wire net (mesh) crack arrestor, and a layer of phenolic resin foam (PRF) attached by aluminium studs to the cryogenic tank wall. All the insulation systems are designed to control the boil-off rate, which is typically in the range of 0.10–0.15%/day.

Self-supporting prismatic tanks are insulated using prefabricated rigid polyurethane foam panels and supported by tank supports and chocks made of specially reinforced wood composites [59]. External rigid PU foam insulation of stationary ground-based cryogenic tanks and LNG tanks exposed to weather conditions is protected with moisture-resistant and UV-protective coatings. 

Much more serious requirements are placed on the insulation of liquid hydrogen tanks. Firstly, the insulation must be cryo-resistant at a lower temperature (the boiling point of hydrogen is 20.4 K instead of the boiling point of methane of 111.6 K). If, at the same time, these are LH_2_ tanks of launch vehicles, aircraft or risible ships, then cryogenic insulation in some parts of the tanks must withstand aerodynamic and, in some cases, even ablative loads [60,61].

External insulation of early LH_2_ tanks of the Saturn V S-II Stage was made of applied phenolic honeycomb panels filled with heat-resistant polyurethane foam of isocyanate. The insulation had a network of passages through which helium gas flowed. In later stages, SOFI was used instead of making up panels and affixing them to the tank. The urethane-based foam was sprayed directly onto the tank walls, letting it cure, then cutting it to the proper contour. This technique turned out to be much more economical and much lighter than the insulation panels [62,63,64,65].

Ripor- and Izolan-sprayed rigid PU foams have been successfully used as cryogenic insulation for liquid hydrogen and oxygen tanks of the insulation of the launcher Energia. The cryogenic thermal insulation based on rigid PU was used for tanks of expendable and reusable launchers [66]. The thermal protection system design of Space Shuttle’s External Tank has changed with changing requirements and improvements in materials and processes. Most changes were directed toward producibility improvements, reduction in recurring costs, and elimination of sole-source dependencies. Increased use of robotics and net-moulding techniques will significantly reduce touch labour and material usage. Different SOFI materials that could potentially reduce the amount of ablator used were tested [67]. As a result, several SOFI were also used to insulate the latest Space Shuttle modifications. PIR foam NCFI 24–124 as acreage foam was sprayed via the automated method onto most of the tank’s surface. Around fittings and flanges, where automated spraying was impossible, the rigid PU foam BX-265 was manually sprayed. For moulded close-outs, rigid PU foam PDL-1034 was used. These materials withstand basic aerodynamic shear and aerodynamic heating at the start and during flight. At the same time, parts of the external tank with the highest aerodynamic heating were additionally protected by ablative materials (materials that gradually erode or vaporise during atmospheric entry, creating a layer of gas that protects spacecraft from extreme heat and pressure). Table 2 lists all used materials in Space Shuttle external tank insulation [68,69,70,71].

Even higher requirements are imposed on the thermal insulation of the cryogenic tanks of the subsonic and hypersonic transport and reusable launch vehicles. Moreover, the main difference is that the thermal insulation of tanks must withstand cryo-shock and aerodynamic loads for a much larger number of cycles than a space rocket. So, to test candidates for subsonic transport, thermal insulation specimens were bonded to a thin, flat aluminium tank. The tests were conducted by filling the tank with liquid hydrogen and exposing the outer surface of the insulation to a cyclic thermal environment representative of repeated subsonic aircraft flights. The boil-off rate in each compartment indicated the thermal performance of the insulation. Two unreinforced polyurethane foams survived 4400 thermal cycles (representative of approximately 15 years of airline service) with evidence of very little structural deterioration [23,72,73].

Operating loads for hydrogen fuel tanks, thermal insulation of supersonic aircraft tanks, and reusable launch vehicles are even higher. Experiments and model calculations show that no single material meets all the requirements. Therefore, various schemes for thermal insulation of cryogenic tanks, consisting of several layers, are offered. Each of them performs its functions. As a rule, PU or polyisocyanurate (PIR) foam performs the functions of cryogenic insulation. Other materials are used as ablative materials [17,74,75,76].

Moreover, we can conclude that cryogenic insulation materials also need to be cryogenic stable but are not only necessary for the coefficient of thermal conductivity. Characteristics such as elongation coefficient of thermal expansion also need to be taken into account. Depending on the construction solution and exploitation conditions, characteristics for strength and other properties can vary. Internal insulation is where exploitation pressure is greater and PU needs to have higher density and can possibly be reinforced with fibres. On the other hand, the external insulation PU foams used could be more lightweight, but it is important to withstand aerodynamic and ablative stresses. It is important to develop insulation for specific use in mind as properties for different insulation types vary.

## 3. Environmentally Friendly Blowing Agents in Cryogenic Rigid Polyurethane Foams

**Figure 1:**
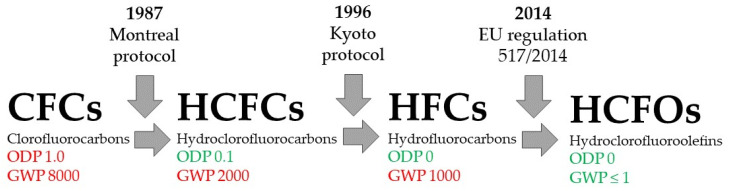
Important milestones in the evolution of physical blowing agents.

Sustainability is becoming more and more important in every field, including cryogenic insulation. There are two possibilities for how to make rigid PU foams more environmentally friendly: blowing agents and polyols. Two types of blowing agents are used in PU production: chemical and physical. Water is used as a chemical blowing agent, and various chemicals with low boiling temperatures are used as a physical blowing agent. Physical blowing agents can expand quickly due to phase changes, such as the vaporisation of liquids. This can reduce overheating caused by isocyanate reaction with polyols’ water and -OH groups. However, most hydrofluorocarbons have high global warming potential (GWP) and ozone depletion potential [77]. Chlorofluorocarbons (CFCs) are so-called first-generation blowing agents. CFCs were banned by the Montreal Protocol in 1987 due to their high ozone-depleting potential and high GWP [77]. The second-generation blowing agents are hydrochlorofluorocarbons (HCFCs). HCFCs were banned by the Kyoto Protocol in 1996 [78,79]. Third-generation blowing agents are hydrofluorocarbons (HFCs). The European Union bans or restricts the usage of fluorinated greenhouse gases with regulation No 517/2014 [80]. This regulation bans or restricts physical blowing agents whose ozone-depleting potential is not 0 and whose global warming potential is higher than 1, which are fourth-generation blowing agents. The evolution of blowing agents is shown in Figure 1.

**Figure 2:**
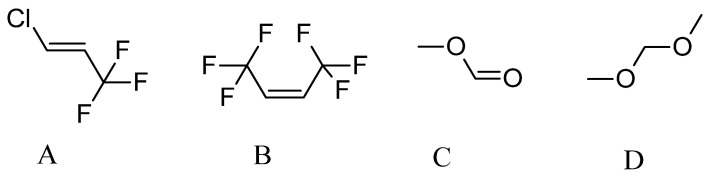
Structural formulas of 4th-generation blowing agents: (**A**) trans-1-chloro-3,3,3-trifluoropropene; (**B**) cis-1,1,1,4,4,4-hexafluoro-2-butene; (**C**) methyl formate; and (**D**) dimethoxymethane.

There are several options for fourth-generation physical blowing agents: trans-1-chloro-3,3,3-trifluoropropene (HFO-1233zd(E), HCFO-1233zd(E), Solstice^®^ LBA), cis-1,1,1,4,4,4-hexafluoro-2-butene (HFO-1336mzz(Z), Opteon^TM^ 1100), dimethoxymethane (Methylal), and methyl formate (R-611; Ecomate^®^) (see Figure 2). Some properties of these physical blowing agents are listed in Table 3 [81,82,83,84]. The most often used physical blowing agents are HFO-1233zd(E) and HFO-1336mzz(Z).

Several studies have been conducted using fourth-generation physical blowing agents in PU foams designed explicitly for cryogenic applications. Yakushin et al. conducted a series of studies about obtaining rigid PU foams with fourth-generation blowing agents [85,86,87]. In the first of the studies, authors compared third-generation physical blowing agents (a blend of 1,1,1,3,3-pentafluorobutane and 1,1,1,2,3,3,3-heptafluoropropane under the trade name Solkane 365/277) with fourth-generation physical blowing agent (HFO-1233zd(E)). The most noticeable difference is that the fourth-generation physical blowing agent has a lower coefficient of thermal conductivity, and rigid PU foams are ageing at a similar speed regardless of the physical blowing agent used in foams. This property ensures a lower coefficient of thermal conductivity over an extended period of time for rigid PU foams containing HFO-1233zd(E) [85].

The second article in this series [86] was dedicated to catalyst selection for rigid PU foams containing HFO-1233zd(E) as a physical blowing agent. Several chemicals are included in a typical catalyst package for rigid PU foam creation. These compounds are meant to enhance either the blowing or gelling process or the balance of both during foam synthesis. Most of these are categorised as “non-reactive” catalysts, which are known to pose a health risk because of their tendency to evaporate and interact with their surroundings. In order to counter this, a number of novel reactive amine catalysts have been created, which, in contrast to their forerunners, react with isocyanate or polyol molecules and integrate into the polymer matrix. The authors tested some new reactive amine catalysts (Polycat 203, Polycat 218, and DABCO MB20) and compared them with more traditional non-reactive catalysts (Polycat 5 and dibutyltin dilaurate). Although some combinations of new catalysts failed in general, rigid PU foam properties (safety coefficient, adhesion strength, and tensile strength) with reactive-type catalysts were higher than traditional non-reactive catalysts [86]. This is important as typically reactive catalysts are less volatile [88,89]. As a result, it could improve working conditions in rigid PU production processes. Also, tin-base catalysts are toxic and raise environmental concerns [90], which is why replacing them can be a slight improvement towards more environmentally friendly rigid PU foams.

The third article in this series focuses on the long-term storage of polyol systems’ component A (polyols + catalysts + blowing agent + flame retardant + surfactant). They compared traditional non-reactive catalysts (Polycat 5) with new reactive amine catalysts (Polycat 203, Polycat 218) in rigid PU systems containing fourth-generation blowing agent HFO-1233zd(E). Certain side reactions occur when polyol mixtures are stored for an extended period of time. These reactions involve the amine catalyst on one side and the halogen-contained blowing agent and flame retardant on the other. These reactions cause the catalyst’s activity and the polyol mixtures’ pH to gradually decrease. Authors observed that rigid PU foams containing non-reactive catalyst foaming parameters (start time and rise time) increase more slowly than reactive amine catalysts. The density of rigid PU foam composition with non-reactive catalyst after long-term storage was higher than the density of rigid PU foam composition with new, specifically made for the fourth-generation blowing agent reactive catalysts, even though this composition with non-reactive catalyst had the shortest foam rise time. After three months of storing the polyol mixture, the non-reactive catalyst caused a noticeable increase in the density of rigid PU foam. Only after storing the polyol mixture with reactive catalysts for four months was the density increase seen [87].

Park et al. compared HFO-1233zd(E) with pentafluoropropane (HFC-245fa) as blowing agents in their rigid PU foams. The authors found that several properties (coefficient of thermal conductivity, compression strength, and coefficient of linear thermal expansion) important for cryogenic applications are slightly higher for rigid PU foams with fourth-generation physical blowing agents [91].

## 4. Bio-Based Polyols in Cryogenic Rigid Polyurethane Foams

More research is conducted every year on rigid PU foams made from bio-based feedstock. Among other biomass, bark and its extracts [92,93,94,95], cellulose [96], suberinic acids [97,98], lignin [99,100,101,102,103,104,105,106], castor oil [107], rice straw [108], soybean oil [109,110], tall oil [111,112,113,114,115,116], and food waste [117] have all been used to make polyols for rigid PU foams. Although plenty of research has been conducted on rigid PU foams as thermal insulation from bio-based polyols, only a few studies have been dedicated to cryogenic insulation. Table 4 shows some available bio-based polyols in the market [118,119].

Several studies from the Latvian State Institute of Wood Chemistry are dedicated to bio-based cryogenic rigid PU foams [120,121]. In all cases, petrochemical raw materials are partially replaced with bio-based and recycled raw materials. Two of those studies are related, in which epoxidised rapeseed and tall oils were used, as well as recycled PET. The first of those studies was dedicated to developing a rigid PU foam system [120]. The second study used the best system and added different fillers [121].

Uram et al. [120] tried replacing diethylene glycol in a rigid PU system with a low-functional rapeseed oil polyol (Table 5). The rigid PU system also contained high-functional rapeseed polyol, epoxidised tall oil–triehylene amine polyol, and recycled polyethylene terephthalate (PET) polyol. The amount of those polyols was kept constant. The authors reached sustainable material content in PU foam at 21.3%, and mechanical properties at room temperature remained similar. However, replacing diethylenel glycol in rigid PU foams significantly worsened cryogenic properties, including thermal conductivity, mechanical properties (tensile and compression), as well as adhesion to aluminium. For example, tensile strength decreased two times with low-functional polyol content increasing from 0 to 20 pbw, and adhesion strength decreased by half with any addition of low-functional polyol. Despite this, the comparison sample had quite good cryogenic properties: thermal conductivity of 18.72 mW/(m·K); compression strength of ~0.5 MPa and ~0.4 MPa parallel and perpendicular to foaming direction, respectively; tensile strength of ~0.95 MPa; and adhesion strength ~0.5 MPa. The authors also used the safety coefficient as a characteristic of cryogenic insulations’ capability to withstand cryogenic stress (Equation (1)); the higher the safety coefficient, the better the material can withstand stress. For cryogenic insulation, the safety coefficient has to be >3.

**Table 5:** Some characteristics of bio-based rigid PU foams from rapeseed and tall oil polyols.

	Components, pbw				
Low-functional rapeseed polyol	0	5	10	15	20
Diethylene glycol	25	20	15	10	5
Sustainable material content in PU foam, %	16.1	17.3	18.7	19.9	21.3
Thermal conductivity, mW/(m·K)	18.7	18.9	20.1	21.4	27.5
Closed-cell content, %	97.5	92.0	91.0	88.0	78.0
Safety coefficient	2.5	1.7	1.1	1.1	1.0

(1) $$k_{S}=\frac{\varepsilon_{77}}{{∆l}_{77-300}}=\frac{\varepsilon_{77}}{\alpha_{x}\cdot ∆T\cdot 100}$$

In Equation (1) above,

ε_77_—tensile elongation at 77 K, %;
Δl_77−300_—shrinkage of material cooling it from 300 to 77 K, %;
α*_x_*—coefficient of thermal expansion, 10^−6^/°C;
ΔT—temperature gradient, degrees.

Sture et al. [121] selected the best Uram et al. [120] rigid PU system and added several silanised and non-silanised fillers: sawdust and micro- and nanocellulose (Table 6). As a result, non-silanised fillers made agglomerates in the PU system; therefore, they were not usable for further testing. Adding fillers increased rigid PU foam density but failed to improve mechanical properties. The authors suggested that more research should be conducted to determine how the cellulose-based filler affects the properties of PU composites and what may be conducted to address poor outcomes, as other authors significantly improved their polymer properties with the addition of fillers [122,123,124].

**Figure 3:**
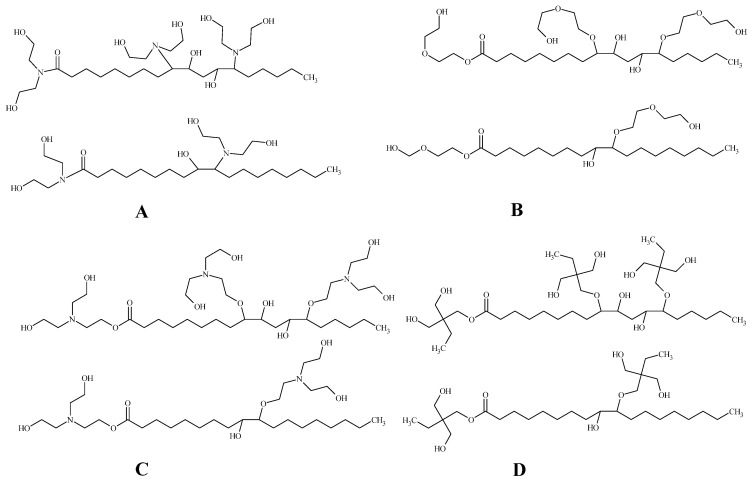
Idealised scheme of the main components of polyols from epoxidised tall oil fatty acids with different ring openers: (**A**) diethanol amine; (**B**) diethylene glycol; (**C**) triethanol amine; and (**D**) trimethylolpropane.

Vevere et al. [125] combined both bio-based and recycled polyols as well as fourth-generation physical blowing agents in their rigid PU foams. They used recycled PET polyol, four different epoxidised tall oil polyols (Figure 3), and two different blowing agents: HFO-1233zd(E) and HFO-1336mzz(Z). This research study focused on tailoring rigid PU foam systems to achieve a foam apparent density of 37–40 kg/m^3^, striking a balance between lightweight properties and mechanical strength. The rigid PU foams obtained exhibited a closed cell structure with elongated cells aligned in the foam rise direction. The morphological features of the cells were influenced by the foaming process and kinetics, including the use of different polyols and blowing agents. The coefficient of thermal conductivity of the rigid PU foams ranged from 17.1 to 21.1 mW/(m·K), comparable to or lower than other bio-based rigid PU foams. Compression strength measurements highlighted the material’s ability to withstand static forces, with values ranging from 0.11 to 0.24 MPa. Tensile strength and adhesion strength properties were also evaluated, showcasing the material’s toughness and bonding capabilities. The safety coefficient, reflecting the material’s ability to endure thermal strains and mechanical stress, exhibited a relation to the mechanical properties of the rigid PU foams. The choice of polyols influenced rigid PU properties minimally as selected polyols had similar properties, which resulted in rigid PU foams with similar cross-link density and properties. On the other hand, the physical blowing agent significantly influenced some properties, such as the coefficient of thermal conductivity and adhesion to aluminium. Rigid PU foams containing HFO-1233zd(E) exhibit notably higher adhesion properties compared to HFO-1336mzz(Z). Rigid PU foams containing HFO-1233zd(E) also had improved thermal conductivity compared to HFO-1336mzz(Z) [125].

## 5. Concluding Remarks

Cryogenic insulation is crucial for any cryogenic technology. Rigid polyurethane foams have been successfully used as cryogenic insulation material for decades. However, moving towards more sustainable cryogenic insulation is still a challenge. Research about using environmentally friendly blowing agents and bio-based and recycled polyols is available but still quite limited.

Currently, scientists have already developed and tested rigid PU foams that contain about 20% of sustainable materials content. Obtained rigid PU foams have a coefficient of thermal conductivity below 20 mW/(m·K) and a safety coefficient above 2, which makes them competitive with rigid PU foams made from fossil-based polyols.

The importance of high-quality and correctly selected cryogenic insulation in future technologies was demonstrated by the successful launch of the European launch vehicle Ariane-6 on 9 July 2024. The main stage and upper stage of Ariane-6 are powered by liquid hydrogen and oxygen, and the fuel tanks were, of course, insulated with effective materials, the properties and development of which are described in this article.

There is still a lot of work to undertake in order to make cryogenic insulation more sustainable.

## Figures and Tables

**Table 1:** Comparison between different types of cryogenic insulation.

Vacuum MLI	Aerogels	Rigid PU Foams
+ Low thermal conductivity	+ Lightweight + Quiet low thermal conductivity	+ Easy to apply + Can cover any shape and form, including connection points + Lightweight + Good mechanical properties + Moisture stable + In the case of damage, it is easily fixed
- Heavier - In the case of damage, it loses insulation properties	- Sensitive to moisture - Often quite fragile	- Some may have problems with cryo-pumping - Higher thermal conductivity than MLI and aerogels

**Table 2:** Space Shuttle external tank insulation.

Material Number	Foam Type	Function	Blowing Agent
**NCFI 24–124**	Polyisocyanurate	Insulation	HCFH 141b
**NCFI 24–57**	Polyisocyanurate	Insulation	HCFH 141b
**BX-250**	Polyurethane	Insulation	CFC-11
**BX-265**	Polyurethane	Insulation	HCFH 141b
**MA-25**	Elastomeric silicone	Ablator	N/A
**SLA-561**	Cork-filled elastomeric silicone	Ablator	N/A
**PDL-1034**	Urethane	Repairs	N/A

**Table 3:** Properties of 4th-generation blowing agents.

Chemical Name	trans-1-Chloro-3,3,3-trifluoropropene	tis-1,1,1,4,4,4-Hexafluoro-2-butene	Dimethoxy Methane	Methyl Formate
CAS number	102687-65-0	692-49-9	109-87-5	107-31-3
Molecular weight, g/mol	130.5	164.1	76.0	60.1
Boiling point, °C	18.6	33.4	42.0	32.0
Freezing point, °C	<−90.0	−90.5	−105.0	−100.0
Global warming potential	1	2	Negligible	0
Ozone depletion potential	0	0	0	0

**Table 4:** Properties of bio-based polyols.

Name	Origin	OHvalue, mgKOH/g	Acid Value, mgKOH/g	Average Functionality
TT	Tall oil	295	<5	2.4
TD	Tall oil	271	1.8	2.2
RD	Rapeseed oil	394	4.8	3.5
GX-9006	Cashew nutshell	201	<0.2	4.4
GX-9101	Cashew nutshell	430	2.8	3.0
GX-9104	Cashew nutshell	235	0.11	3.0
NX-9001	Cashew nutshell	193	<0.2	4.3
NX-9004	Cashew nutshell	193	<0.2	3.8

**Table 6:** Some characteristics of bio-based rigid PU foams from rapeseed and tall oil polyols with fillers.

		Components, pbw		
Filler	-	Sawdust	Microcelullose	Nanocellulose
Sustainable material content in PU foam, %	n.a.	n.a.	n.a.	n.a.
Thermal conductivity, mW/(m·K)	18.7	17.7	18.5	18.1
Closed-cell content, %	n.a.	n.a.	n.a.	n.a.
Safety coefficient	2.7	2.2		2.3

## References

[1] DIRECTIVES DIRECTIVE (EU) 2018/2001 OF THE EUROPEAN PARLIAMENT AND OF THE COUNCIL of 11 December 2018 on the Promotion of the Use of Energy from Renewable Sources. https://eur-lex.europa.eu/legal-content/EN/TXT/PDF/?uri=CELEX:32018L2001.

[2] Long-Term Aspirational Goal Overview of Climate Goals and ICAO’s Work on a Long-Term Aspirational Goal for International Aviation (LTAG) Overview of Climate Goals and ICAO’s Work on a Long-Term Aspirational Goal for International Aviation (LTAG) by ICAO Secretariat. https://www.icao.int/environmental-protection/Documents/EnvironmentalReports/2022/ENVReport2022_Special%20Supplement%20on%20LTAG.pdf.

[3] Fesmire J.E. (2015). Standardization in Cryogenic Insulation Systems Testing and Performance Data. Phys. Procedia.

[4] Johnson W., Fesmire J. (2012). Thermal Performance of Low Layer Density Multilayer Insulation Using Liquid Nitrogen. AIP Conf. Proc..

[5] Xie G.F., Li X.D., Wang R.S. (2010). Study on the Heat Transfer of High-Vacuum-Multilayer-Insulation Tank after Sudden, Catastrophic Loss of Insulating Vacuum. Cryogenics.

[6] Sun P.J., Wu J.Y., Zhang P., Xu L., Jiang M.L. (2009). Experimental Study of the Influences of Degraded Vacuum on Multilayer Insulation Blankets. Cryogenics.

[7] Zheng J., Chen L., Cui C., Guo J., Zhu W., Zhou Y., Wang J. (2018). Experimental Study on Composite Insulation System of Spray on Foam Insulation and Variable Density Multilayer Insulation. Appl. Therm. Eng..

[8] Jiang W., Yang Y., Hu C., Li P., Sun P., Huang Y. (2023). Experimental Study on Composite Insulation with Foam, Multilayer and Vapor Cooled Shield for Cryogen Storage under Different Vacuum Conditions. Cryogenics.

[9] Ye C., Lin Y., Pei F. (2022). Comparative Study of Three Insulation Materials Installed on Type C Independent Tank for Offshore LNG Transportation. Cryogenics.

[10] Fesmire J.E., Ancipink J.B., Swanger A.M., White S., Yarbrough D. (2017). Thermal Conductivity of Aerogel Blanket Insulation under Cryogenic-Vacuum Conditions in Different Gas Environments. IOP Conference Series: Materials Science and Engineering, Proceedings of the Cryogenic Engineering Conference (CEC) 2017 (Previous Edition: CEC-2015), Madison, WI, USA, 9–13 July 2017.

[11] Global Hydrogen Review 2023. https://www.iea.org/reports/global-hydrogen-review-2023.

[12] Ustolin F., Campari A., Taccani R. (2022). An Extensive Review of Liquid Hydrogen in Transportation with Focus on the Maritime Sector. J. Mar. Sci. Eng..

[13] Adler E.J., Martins J.R.R.A. (2023). Hydrogen-Powered Aircraft: Fundamental Concepts, Key Technologies, and Environmental Impacts. Prog. Aerosp. Sci..

[14] Verstraete D., Hendrick P., Pilidis P., Ramsden K. (2010). Hydrogen Fuel Tanks for Subsonic Transport Aircraft. Int. J. Hydrogen Energy.

[15] Winnefeld C., Kadyk T., Bensmann B., Krewer U., Hanke-Rauschenbach R. (2018). Modelling and Designing Cryogenic Hydrogen Tanks for Future Aircraft Applications. Energies.

[16] Huete J., Pilidis P. (2021). Parametric Study on Tank Integration for Hydrogen Civil Aviation Propulsion. Int. J. Hydrogen Energy.

[17] Kameni Monkam L., Graf von Schweinitz A., Friedrichs J., Gao X. (2022). Feasibility Analysis of a New Thermal Insulation Concept of Cryogenic Fuel Tanks for Hydrogen Fuel Cell Powered Commercial Aircraft. Int. J. Hydrogen Energy.

[18] Alkhaledi A.N.F.N.R., Sampath S., Pilidis P. (2022). A Hydrogen Fuelled LH2 Tanker Ship Design. Ships Offshore Struct..

[19] Kang D., Yun S., Kim B.K. (2022). Review of the Liquid Hydrogen Storage Tank and Insulation System for the High-Power Locomotive. Energies.

[20] Lebrun P. Introduction to Cryogenics Cryogenic Fluids. Proceedings of the CAS 2006—CERN Accelerator School: Vacuum in Accelerators, Proceedings.

[21] Daunt J.G. (1962). Cryogenics. Phys. Today.

[22] International Institute of Refrigeration International Dictionary of Refrigeration. https://dictionary.iifiir.org/index.php.

[23] Anthony F.M., Colt J.Z., Helenbrook R.G. (1981). Development and Validation of Cryogenic Foam Insulation for LH2 Subsonic Transports.

[24] Peschka W. (1992). Thermal Insulation, Storage and Transportation of Liquid Hydrogen. Liquid Hydrogen.

[25] Colozza A.J. (2002). Hydrogen Storage for Aircraft.

[26] Musgrave D.S. (1979). Thermal Performance of Urethane Pipe Insulation at Cryogenic Temperatures. J. Therm. Insul..

[27] Darkow N., Fischer A., Scheufler H., Hellmann H., Gerstmann J. Concept Development of a Cryogenic Tank Insulation for Reusable Launch Vehicle. Proceedings of the 8th European Conference for Aeronautics and Space Sciences (EUCASS).

[28] Sullivan R.M., Palko J.L., Tornabene R.T., Bednarcyk B.A., Powers L.M., Smith L.M., Wang X.J., Hunter J.E. (2006). Engineering Analysis Studies for Preliminary Design of Lightweight Cryogenic Hydrogen Tanks in UAV Applications.

[29] Mital S.K., Gyekenyesi J.Z., Arnold S.M., Sullivan R.M., Manderscheid J.M., Murthy P.L.N. (2006). Review of Current State of the Art and Key Design Issues with Potential Solutions for Liquid Hydrogen Cryogenic Storage Tank Structures for Aircraft Applications.

[30] Ten C.L. (1974). Advances in Heat Transfer.

[31] Fesmire J.E., Coffman B.E., Meneghelli B.J., Heckle K.W. (2012). Spray-on Foam Insulations for Launch Vehicle Cryogenic Tanks. Cryogenics.

[32] Tien C.L., Cunnington G.R. (1976). Glass Microsphere Cryogenic Insulation. Cryogenics.

[33] Ratnakar R.R., Sun Z., Balakotaiah V. (2023). Effective Thermal Conductivity of Insulation Materials for Cryogenic LH2 Storage Tanks: A Review. Int. J. Hydrogen Energy.

[34] Dearing D.L. (1966). Development of the Saturn S-IV and S-IVB Liquid Hydrogen Tank Internal Insulation. Advances in Cryogenic Engineering.

[35] Bauer H.E. (1968). Operational Experiences on the Saturn V S-IVB Stage.

[36] Lemons C.R., Watts C.R., Salmassy O.K. (2019). Development of Advanced Materials Composites for Use as Insulations LH2 Tanks. Angew. Chem. Int. Ed..

[37] Li K., Wu Z., Liu M., Xu X., Xu W. (2020). An Application of Reinforced Polyurethane Foam in Design of the Common Bulkhead for Cryogenic Tanks. Mater. Sci. Forum.

[38] Lee D.H., Ha M.K., Kim S.Y., Shin S.C. (2014). Research of Design Challenges and New Technologies for Floating LNG. Int. J. Nav. Archit. Ocean. Eng..

[39] Oh D.J., Lee J.M., Chun M.S., Kim M.H. (2017). Reliability Evaluation of a LNGC Insulation System with a Metallic Secondary Barrier. Compos. Struct..

[40] Huan T., Hongjun F., Wei L., Guoqiang Z. (2019). Options and Evaluations on Propulsion Systems of LNG Carriers. Propulsion Systems.

[41] Kim J.H., Kim S.K., Kim M.S., Lee J.M. (2014). Numerical Simulation of Membrane Og LNG Insulation System Using User Defined Material Subroutine. J. Comput. Struct. Eng. Inst. Korea.

[42] Hwang B.K., Kim S.K., Kim J.H., Kim J.D., Lee J.M. (2020). Dynamic Compressive Behavior of Rigid Polyurethane Foam with Various Densities under Different Temperatures. Int. J. Mech. Sci..

[43] Yu Y.H., Choi I., Nam S., Lee D.G. (2014). Cryogenic Characteristics of Chopped Glass Fiber Reinforced Polyurethane Foam. Compos. Struct..

[44] Park S.B., Choi S.W., Kim J.H., Bang C.S., Lee J.M. (2016). Effect of the Blowing Agent on the Low-Temperature Mechanical Properties of CO2- and HFC-245fa-Blown Glass-Fiber-Reinforced Polyurethane Foams. Compos. B Eng..

[45] Song C.Y., Cho D.Y. (2018). Cryogenic Compressive Strength and Thermal Deformation of Reinforced Polyurethane Foam Material for Membrane Type LNG Carrier. Proceedings of the Key Engineering Materials.

[46] Song H.-C. (2022). Assessment of Cryogenic Material Properties of R-PUF Used in the CCS of an LNG Carrier. J. Ocean. Eng. Technol..

[47] Kim J.D., Kim J.H., Lee D.H., Yeom D.J., Lee J.M. (2021). Synthesis and Investigation of Cryogenic Mechanical Properties of Chopped-Glass-Fiber-Reinforced Polyisocyanurate Foam. Materials.

[48] Park S.B., Lee C.S., Choi S.W., Kim J.H., Bang C.S., Lee J.M. (2016). Polymeric Foams for Cryogenic Temperature Application: Temperature Range for Non-Recovery and Brittle-Fracture of Microstructure. Compos. Struct..

[49] Kim J.-H., Kim S.-K., Park S., Hyun Park K., Lee J.-M. (2017). Low-Temperature Mechanical Behavior of Reinforced Polyurethane Foam. Int. J. Mech. Eng..

[50] Park K.B., Kim H.T., Her N.Y., Lee J.M. (2019). Variation of Mechanical Characteristics of Polyurethane Foam: Effect of Test Method. Materials.

[51] Chun M.S., Kim M.H., Kim W.S., Kim S.H., Lee J.M. (2009). Experimental Investigation on the Impact Behavior of Membrane-Type LNG Carrier Insulation System. J. Loss Prev. Process Ind..

[52] Yu Y.H., Nam S., Lee D., Lee D.G. (2015). Cryogenic Impact Resistance of Chopped Fiber Reinforced Polyurethane Foam. Compos. Struct..

[53] Oh J.H., Bae J.H., Kim J.H., Lee C.S., Lee J.M. (2019). Effects of Kevlar Pulp on the Enhancement of Cryogenic Mechanical Properties of Polyurethane Foam. Polym. Test..

[54] Kim J.H., Kim J.M., Park S., Park K.H., Lee J.M. (2018). Synthesis and Cryogenic Mechanical Properties of CO2-Blown Carbon-Reinforced Polyurethane Foam. J. Cell. Plast..

[55] Park J.H., Oh D.J., Kim M.H., Kim K.H., Kim M.K., Moon H.S. (2018). Fatigue Strength of a LNGC Secondary Barrier Made of a Composite Material with Aramid Fibers. Mech. Compos. Mater..

[56] Yu Y.H., Kim B.G., Lee D.G. (2013). Cryogenic Reliability of the Sandwich Insulation Board for LNG Ship. Compos. Struct..

[57] Yu Y.H., Kim B.G., Lee D.G. (2012). Cryogenic Reliability of Composite Insulation Panels for Liquefied Natural Gas (LNG) Ships. Compos. Struct..

[58] Lee C.S., Lee J.M. (2014). Failure Analysis of Reinforced Polyurethane Foam-Based LNG Insulation Structure Using Damage-Coupled Finite Element Analysis. Compos. Struct..

[59] Soumya Chakraborty Understanding the Design of Liquefied Gas Carriers. https://www.marineinsight.com/naval-architecture/understanding-design-liquefied-gas-carriers/.

[60] Giannopoulos I.K., Theotokoglou E.E. (2024). Liquid Hydrogen Storage Tank Loading Generation for Civil Aircraft Damage Tolerance Analysis. Proceedings of the Journal of Physics: Conference Series.

[61] Yatsenko E.A., Goltsman B.M., Novikov Y.V., Izvarin A.I., Rusakevich I.V. (2022). Review on Modern Ways of Insulation of Reservoirs for Liquid Hydrogen Storage. Int. J. Hydrogen Energy.

[62] Mack F.E., Smith M.E. High-Performance Spray-Foam Insulation for Application on Saturn S-II Stage*. https://ntrs.nasa.gov/citations/19710058573.

[63] McCutcheon K.D. U.S. Manned Rocket Propulsion Evolution. Part 8.20: The Saturn V S-II Stage. https://www.enginehistory.org/Rockets/RPE08.20/RPE08.20.shtml.

[64] Mike Jetzer The Apollo Flight Journal S-II Insulation. https://www.nasa.gov/history/afj/s-ii/s-ii-insulation.html.

[65] Bilstein R.E. Stages to Saturn: A Technological History of the Apollo/Saturn Launch Vehicles. NASA SP-4206. https://ntrs.nasa.gov/citations/19970009949.

[66] Gusev Y., Kolozezny A., Panichkin N., Volkov N. (2005). Cryogenic Thermal Insulation Based on PUF for Tank…. Proceedings of the 56th International Astronautical Congress of the International Astronautical Federation, the International Academy of Astronautics, and the International Institute of Space Law.

[67] Gray C., Ronquillo L., Williams C. (1984). Spray-on Foam Insulation Development for the Space Shuttle External Tank Thermal Protection System.

[68] Gates T.S., Johnson T.F., Whitley K.S. (2005). Assessment of Technologies for the Space Shuttle External Tank Thermal Protection System and Recommendations for Technology Improvement Part 3: Material Property Characterization, Analysis, and Test Methods.

[69] Columbia Accident Investigation Board Report Volume 1. Chapter 3. Accident Analysis. https://www.globalsecurity.org/space/library/report/2003/caib-report_vol1_chapter3.pdf.

[70] Sullivan R.M., Lerch B.A., Rogers P.R., Sparks J.S. An Overview of Spray-On Foam Insulation Applications on the Space Shuttle’s External Tank: Foam Applications and Foam Shedding Mechanisms; Mechanisms 43rd Annual Technical Meeting of the Society of Engineering Science 2006. https://ntrs.nasa.gov/api/citations/20070008105/downloads/20070008105.pdf.

[71] Dreggors K. (2005). Alternative Foam Treatments for The Space Shuttle’s External Tank. https://stars.library.ucf.edu/etd/548/?utm_source=stars.library.ucf.edu%2Fetd%2F548&utm_medium=PDF&utm_campaign=PDFCoverPages.

[72] Sharpe E.L., Clark A.F., Reed R.P., Hartwig G. (1979). Durability of Foam Insulation for LH2 Fuel Tanks of Future Subsonic Transports. Nonmetallic Materials and Composites at Low Temperatures.

[73] Cunnington G.R., Lockheed Palo J.R. (1980). Insulation Systems for Liquid Hydrogen Fueled Aircraft. J. Therm. Insul..

[74] Brewer G.D. (2017). Hydrogen Aircraft Technology.

[75] Wilken J., Callsen S., Daub D., Fischer A., Liebisch M., Rauh C., Reimer T., Scheufler H., Sippel M. Combined Cryogenic Insulation and Thermal Protection Systems for Reusable Stages. Proceedings of the 9th European Conference for Aeronautics and Space Sciences (EUCASS).

[76] Sharifzadeh S., Verstraete D., Hendrick P. (2015). Cryogenic Hydrogen Fuel Tanks for Large Hypersonic Cruise Vehicles. Int. J. Hydrogen Energy.

[77] The Montreal Protocol. https://www.unep.org/ozonaction/who-we-are/about-montreal-protocol.

[78] The Kyoto Protocol. https://unfccc.int/kyoto_protocol.

[79] Poulopoulos S.G. (2016). Atmospheric Environment. Environment and Development.

[80] Regulation (EU) No. 517/2014 of the European Parliament and of the Council on Fluorinated Greenhouse Gases and Repealing Regulation (EC) No. 842/2006. https://www.fao.org/faolex/results/details/en/c/LEX-FAOC133686/#:~:text=European%20Union-,Regulation%20(EU)%20No.,emissions%20of%20fluorinated%20greenhouse%20gases.

[81] Methylal as Blowing Agent in the Manufacture of Polyurethane Foam Systems. http://multilateralfund.org/Our%20Work/DemonProject/Document%20Library/6617p5%20methylal%20PU%20foam.pdf.

[82] Opteon TM 1100 Foam Blowing Agent Product Information. https://www.opteon.com/en/-/media/files/opteon/opteon-1100-data-sheet.pdf?la=en&.

[83] Blowing Agents Solstice Liquid Blowing Agent TECHNICAL INFORMATION. https://prod-edam.honeywell.com/content/dam/honeywell-edam/pmt/oneam/en-us/blowing-agents/documents/pmt-am-solstice-lba-datasheet.pdf.

[84] Ecomate. https://ecomatetechnology.com/.

[85] Yakushin V., Cabulis U., Fridrihsone V., Kravchenko S., Pauliks R. (2021). Properties of Polyurethane Foam with Fourth-Generation Blowing Agent. E-Polymers.

[86] Yakushin V., Rundans M., Holynska M., Sture B., Cabulis U. (2023). Influence of Reactive Amine-Based Catalysts on Cryogenic Properties of Rigid Polyurethane Foams for Space and On-Ground Applications. Materials.

[87] Sture B., Yakushin V., Vevere L., Cabulis U. (2023). Influence of Long-Term Storage and UV Light Exposure on Characteristics of Polyurethane Foams for Cryogenic Insulation. Materials.

[88] Casati F.M., Sonney J.M., Mispreuve H., Fanget A., Herrington R., Tu J. (2020). Elimination of Amine Emissions from Polyurethane Foams: Challenges and Opportunities. Polyurethanes Expo 2001.

[89] Sikorski M., Wehman C., Cordelair H. (2000). New Additive Solutions for Low VOC in HR Molded Foams. J. Cell. Plast..

[90] Pretti C., Oliva M., Mennillo E., Barbaglia M., Funel M., Reddy Yasani B., Martinelli E., Galli G. (2013). An Ecotoxicological Study on Tin- and Bismuth-Catalysed PDMS Based Coatings Containing a Surface-Active Polymer. Ecotoxicol. Environ. Saf..

[91] Park J.S., Kim H.T., Kim J.D., Kim J.H., Kim S.K., Lee J.M. (2022). Eco-Friendly Blowing Agent, HCFO-1233zd, for the Synthesis of Polyurethane Foam as Cryogenic Insulation. J. Appl. Polym. Sci..

[92] Vevere L., Arshanitsa A., Telysheva G. The Oxypropylation of Residual Grey Alder Bark as a Tool for Realisation of Biorefinery Concept. https://alephfiles.rtu.lv/TUA01/000047431_e.pdf.

[93] Arshanitsa A., Ponomarenko J., Pals M., Jashina L. (2023). Controlling the Reactivity of Hydrophilic Bark Extractives as Biopolyols in Urethane-Formation Reactions Using Various Catalysts. Ind. Crops Prod..

[94] Arshanitsa A., Ponomarenko J., Pals M., Jashina L., Lauberts M. (2023). Impact of Bark-Sourced Building Blocks as Substitutes for Fossil-Derived Polyols on the Structural, Thermal, and Mechanical Properties of Polyurethane Networks. Polymers.

[95] Vevere L., Janceva S., Arshanitsa A., Telysheva G. (2018). Polyols from Condensed Tannin Enriched Extracts for Rigid Polyurethane Foam. Key Eng. Mater..

[96] Olszewski A., Kosmela P., Vēvere L., Kirpluks M., Cabulis U., Piszczyk Ł. (2024). Effect of Bio-Polyol Molecular Weight on the Structure and Properties of Polyurethane-Polyisocyanurate (PUR-PIR) Foams. Sci. Rep..

[97] Ivdre A., Kirpluks M., Abolins A., Vevere L., Sture B., Paze A., Godina D., Rizikovs J., Cabulis U. (2024). Rigid Polyurethane Foams’ Development and Optimization from Polyols Based on Depolymerized Suberin and Tall Oil Fatty Acids. Polymers.

[98] Ivdre A., Abolins A., Volkovs N., Vevere L., Paze A., Makars R., Godina D., Rizikovs J. (2023). Rigid Polyurethane Foams as Thermal Insulation Material from Novel Suberinic Acid-Based Polyols. Polymers.

[99] Mazar A., Paleologou M. (2023). New Approach to Recycle and Valorize the First Filtrate of the LignoForce System^TM^: Lignin Extraction and Its Use in Rigid Lignin-Based Polyurethane Foams. Int. J. Biol. Macromol..

[100] Du J., Wang H., Huang Z., Liu X., Yin X., Wu J., Lin W., Lin X., Yi G. (2023). Construction and Mechanism Study of Lignin-Based Polyurethane with High Strength and High Self-Healing Properties. Int. J. Biol. Macromol..

[101] Huang Z., Wang H., Du J., Liu X., Pan G., Yin X., Lin W., Lin X., Sun Y., Yi G. (2023). High-Strength, Self-Reinforcing and Recyclable Multifunctional Lignin-Based Polyurethanes Based on Multi-Level Dynamic Cross-Linking. Chem. Eng. J..

[102] Ajao O., Benali M., Faye A., Li H., Maillard D., Ton-That M.T. (2021). Multi-Product Biorefinery System for Wood-Barks Valorization into Tannins Extracts, Lignin-Based Polyurethane Foam and Cellulose-Based Composites: Techno-Economic Evaluation. Ind. Crops Prod..

[103] Wang J., Yao K., Korich A.L., Li S., Ma S., Ploehn H.J., Iovine P.M., Wang C., Chu F., Tang C. (2011). Combining Renewable Gum Rosin and Lignin: Towards Hydrophobic Polymer Composites by Controlled Polymerization. J. Polym. Sci. A Polym. Chem..

[104] Arshanitsa A., Vevere L., Telysheva G., Dizhbite T., Gosselink R.J., Bikovens O., Jablonski A. (2015). Functionality and Physico-Chemical Characteristics of Wheat Straw Lignin, Biolignin^TM^, Derivatives Formed in the Oxypropylation Process. Holzforschung.

[105] Arshanitsa A., Paberza A., Vevere L., Cabulis U., Telysheva G. (2014). Two Approaches for Introduction of Wheat Straw Lignin into Rigid Polyurethane Foams. Proceedings of the AIP Conference Proceedings.

[106] Kurańska M., Pinto J.A., Salach K., Barreiro M.F., Prociak A. (2020). Synthesis of Thermal Insulating Polyurethane Foams from Lignin and Rapeseed Based Polyols: A Comparative Study. Ind. Crops Prod..

[107] Lavazza J., Zhang Q., de Kergariou C., Comandini G., Briscoe W.H., Rowlandson J.L., Panzera T.H., Scarpa F. (2024). Rigid Polyurethane Foams from Commercial Castor Oil Resins. Polym. Test..

[108] Samranrit T., Ngernsombat K., Ritthisorn S., Teeka J., Chiu C.H., Reungsang A., Areesirisuk A. (2024). Sustainable Production of Yeast Oil from Rice Straw Hydrolysate by Pseudozyma Parantarctica through Fed-Batch Cultivation for Bio-Polyurethane Foam Formation. Bioresour. Technol. Rep..

[109] Luo X., Xiao Y., Wu Q., Zeng J. (2018). Development of High-Performance Biodegradable Rigid Polyurethane Foams Using All Bioresource-Based Polyols: Lignin and Soy Oil-Derived Polyols. Int. J. Biol. Macromol..

[110] Fan X., Wang H., Kong L., Huang J. (2024). Advanced Ethylene-Absorbing and Cushioning Depending on the 3D Porous-Structured Fruit Packaging: Toward Polyurethane Foam Manipulation Using Zein and Soybean Oil Polyol Substrates. Food Res. Int..

[111] Ivdre A., Soto G.D., Cabulis U. (2016). Polyols Based on Poly(Ethylene Terephthalate) and Tall Oil: Perspectives for Synthesis and Production of Rigid Polyurethane Foams. J. Renew. Mater..

[112] Pietrzak K., Kirpluks M., Cabulis U., Ryszkowska J. (2014). Effect of the Addition of Tall Oil-Based Polyols on the Thermal and Mechanical Properties of Ureaurethane Elastomers. Polym. Degrad. Stab..

[113] Polaczek K., Kaulina E., Pomilovskis R., Fridrihsone A., Kirpluks M. (2022). Epoxidation of Tall Oil Fatty Acids and Tall Oil Fatty Acids Methyl Esters Using the SpinChem^®^ Rotating Bed Reactor. J. Polym. Environ..

[114] Vanags E., Kirpluks M., Cabulis U., Walterova Z. (2018). Highly Functional Polyol Synthesis from Epoxidized Tall Oil Fatty Acids. J. Renew. Mater..

[115] Abolins A., Pomilovskis R., Vanags E., Mierina I., Michalowski S., Fridrihsone A., Kirpluks M. (2021). Impact of Different Epoxidation Approaches of Tall Oil Fatty Acids on Rigid Polyurethane Foam Thermal Insulation. Materials.

[116] Andersons J., Grūbe R., Vēvere L., Cābulis P., Kirpluks M. (2022). Anisotropic Thermal Expansion of Bio-Based Rigid Low-Density Closed-Cell Polyurethane Foams. J. Mater. Res. Technol..

[117] Qin Z.H., Fridrihsone A., Mou J.H., Pomilovskis R., Godina D., Miao Y., Liu Z., Tsang C.W., Zhang L., Xu C. (2024). Valorisation of Food Waste into Bio-Based Polyurethane Rigid Foams: From Experimental Investigation to Techno-Economic Analysis. Chem. Eng. J..

[118] Cardolite Inc. Home Page. https://www.cardolite.com/.

[119] Polylabs Ltd. Home Page. https://www.polylabs.eu/.

[120] Uram K., Prociak A., Vevere L., Pomilovskis R., Cabulis U., Kirpluks M. (2021). Natural Oil-Based Rigid Polyurethane Foam Thermal Insulation Applicable at Cryogenic Temperatures. Polymers.

[121] Sture B., Vevere L., Kirpluks M., Godina D., Fridrihsone A., Cabulis U. (2021). Polyurethane Foam Composites Reinforced with Renewable Fillers for Cryogenic Insulation. Polymers.

[122] Yakushin V., Stirna U., Bel’kova L., Deme L., Sevastyanova I. (2011). Properties of Rigid Polyurethane Foams Filled with Milled Carbon Fibers. Mech. Compos. Mater..

[123] Moon R.J., Martini A., Nairn J., Simonsen J., Youngblood J. (2011). Cellulose Nanomaterials Review: Structure, Properties and Nanocomposites. Chem. Soc. Rev..

[124] Siró I., Plackett D. (2010). Microfibrillated Cellulose and New Nanocomposite Materials: A Review. Cellulose.

[125] Vevere L., Sture B., Yakushin V., Kirpluks M., Cabulis U. (2024). Bio-Based Rigid Polyurethane Foams for Cryogenic Insulation. J. Renew. Mater..

